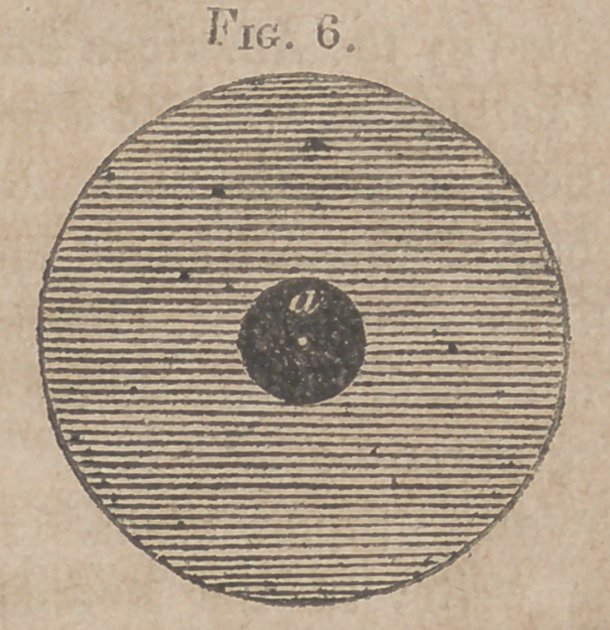# A Treatise on the Diseases of the Chest, in Which They Are Described According to Their Anatomical Characters; and Their Diagnosis Is Established on a New Principle by Means of Acoustick Instruments, with Plates

**Published:** 1829

**Authors:** 


### REVIEWS AND BIBLIOGRAPHICAL NOTICES.

---

ART. VIII.—*A Treatise on the Diseases of the Chest, in which they are described according to their anatomical characters; and their Diagnosis is established on a new principle by means of Acoustick instruments, with plates. Translated from the French of* R. T. H. Laennec, M. D. *with a preface and notes,* By JOHN FORBES, M. D. *&c. First American Edition, Philadelphia,* 1823. *8vo. pp.* 819.

*A short Treatise on the different methods of investigating the Diseases of the Chest. Translated from the French of* M. Collin. By W. N. RYLAND, M. D. *From the third London edition; with plates and an explanatory introduction by a Fellow of the Massachusetts Medical Society. Boston,* 1829. 12*mo. pp.* 133.

If we have passed through more than two years of a *sort* of editorial life, without having presented to our readers the discoveries and inventions of Laennec, it has not been because we thought them of no value. On the contrary, after deducting all the advantages which enthusiasm has superadded to the catalogue, we still find enough left to fix the attention of the profession. To what extent that attention has already been directed upon this subject we are not fairly prepared to say; but of this we are convinced, that neither in Great Britain nor America, have the labors of that distinguished physician been duly appreciated. Having at length taken up the subject we propose to prosecute it in the present and future numbers of the Western Journal, until all the important facts which relate to it, shall be fairly set forth.

Besides many vital and chemical processes, the THORAX is the seat of two mechanical operations, the one *pneumatic* the other *hydraulic*—the former executed by the lungs and parieties of the chest, the latter by the heart. In the present number for the sake of simplicity we shall confine ourselves to the pneumatic.

The object of this function is the æreation of the blood. To accomplish this great end, so immediately indispensable to the continuance of all the other functions, a large quantity of air must be incessantly introduced into, and expelled from, the immense congeries of cells which make up the pneumatic portion of the pulmonary organ. In healthy states of this organ the phenomena attendant on this operation, present much uniformity of character, but performed in a cavity the sides of which are thick and bony, they generally pass unobserved. When either the lungs or the thoracic walls become disordered, these phenomena are of necessity altered, and this departure comprehends the signs of pulmonary disease. Till lately, the nature and degree of these aberrations from health, were measured by the pain and dyspnœa, the cough and expectoration, the posture of the patient, the absence or presence of tenderness on pressure, and by the pulse—methods which can never be abandoned for other means of investigation. It did not follow, however, that other means of diagnosis might not be superadded, and this is precisely what is attempted in the works which constitute the text of this article.

To the organs of touch and vision, we are indebted for the information collected on the old method, that furnished by the new, depends on the sense of hearing. The first step in this diagnostic method was made by Avenbrugger, the second and greater, by Laennec. By *Percussion* over the thoracic parieties, first palpably recommended by the former, we come to know, that in a healthy state, the chest emits on being struck, a peculiar sound, which is either changed, reduced or suppressed in its diseased conditions. By the *Auscultation* or listening of Laennec, we learn that the admission and expulsion of air into the pulmonary cells, and its vocal agitations in the larynx, can be heard and appreciated in health, and, consequently in disease—when the sounds emitted, are of necessity, more or less widely different from those ordinarily heard.

We will proceed to inquire into these natural accoustic phenomena.

First of Percussion. When the lungs and parieties are healthy the chest struck returns a hollow sound not unlike that emitted by an empty barrel, though generally more obtuse. Every part of the thorax does not give out the same sound.

A clear sound is obtained whenever we strike upon a bony part covered by the skin only, or by thin muscles sufficiently stretched to transmit the shock wholly, without absorbing any of the sound.—The most favourable parts are, anteriorly, the clavicles, when they are not too much elevated and raised from the thorax; the space lying two or three inches below them; all the surface of the sternum, and the parts nearest the cartilages of the ribs.

In the remaining anterior part of the thorax, the mamma in females, the fat which covers over the middle and inferior part of the pectoralis major in many men, the vicinity of the liver on the right, and of the heart on the left, diminish the sonorousness natural to the thorax.

Upon the sides we may strike with advantage, in the hollow of the axilla, and three inches below it; but from the fourth and sometimes the third rib downwards, the sound is always less clear on the right, owing to the neighborhood of the liver; while on the left, it is often louder than it ought to be from the proximity of the stomach, particularly when that viscus is much distended by air; the resonance then becomes something metallic.

Behind, the most distinct sound is perceived along the costal angles. In thin persons we may percuss usefully on the supra and infraspinal hollows of the scapulæ, and upon the spine of that bone, but we cause no sound in striking upon the fleshy layer of muscles which fills up the vertebral hollows.—Collin, pp. 14—16.

If the subject be fat, or the integuments anasarcous, the sound *cæteris paribus*, is duller and weaker. The patient at the time of percussing him, should not be sunk in feathers, for this will vary the result. If possible, he should be seated with his arms raised over his head; or, if he lies down, he should be stretched on his back, upon a hard and level bed, with his arms in the position just mentioned. If he makes a deep inspiration and then holds his breath, the sound will be more intense.

On the manner of performing the operation, Collin has the following remarks, which should be read with attention.

We should close and bring together all the ends of the fingers half bent, or we should form them upon the same line, so that one may not project beyond the other. We should then strike with an equal and moderate force upon similar parts, in the same direction and extent, making the ends of the fingers fall perpendicularly to the plane upon which we strike.

Too strong percussion would be painful; an unequal one would give an unsatisfactory result. It would be the same if we struck dissimilar parts, as a rib and an intercostal space; if the fingers inclined perpendicularly on the right, obliquely on the left side; on a space double or triple the extent on one side to the other, since each of these variations must necessarily modify the sound. It is also necessary not to examine all the points of one side before passing to the other, because we thus lose the remembrance of the results obtained in corresponding points; it is better to strike first on one side, and then on the other.

To fulfil all these conditions, none of which are superfluous, we must strike as much as possible with the same hand, and place it in the same direction relatively to the part struck.—pp. 17—18.

In the analectic department of a preceding number of this journal, we published a notice of an alledged improvement in percussing, made by Piorri. It consists in directing the blows upon a circular piece of ivory or wood, an inch and a half in diameter and one line in thickness, having a slender handle to keep it properly applied to the surface. It may be struck with any kind of small mallet. If this plate be used, percussion we are told may be made over the clothes. We have not ourselves employed it.

In diseases of the chest, the hollow sound returned by percussion is altered. It may be dull and obscure, entirely suppressed, or unnaturally clear.

Every time the lung loses its elasticity and becomes engorged, without however becoming totally impermeable, the sound becomes dull or obscure, according as the engorging of the pulmonary tissue is more or less considerable. Thus then an intense catarrh, bronchitis, the first degree of pneumonia, an œdema of the lung, produce this alteration.

The sound becomes wanting in two cases; 1st. When the lung loses its permeability completely, its tissue becoming dense, like the substance of the liver, in consequence of an abundant exhalation of blood into its areolæ, and of the combination of this liquid with its tissue. 2d. When it is compressed, thrust back by some accidental production, developed in its thickness, or in the cavity of the pleura, [illegible] when this cavity is filled by some fluid.—p. 20.

When the hollow sound is entirely suppressed, the noise under percussion, as Laennec has remarked, is like that emitted by the thigh or any solid, fleshy part when struck. The finger perceives, that there is no longer any elasticity in the parieties nor an empty cavity within. This can never occur over the whole thorax; for it implies a state of the parts incompatible with the function of respiration. It occurs chiefly in the lower half of the chest, and is oftener perceived on the right than on the left side, because the liver as we have already quoted, renders the inferior portion of that side, more inelastic than any other part; while, on the other hand, the stomach so often inflated with gases, renders the opposite side unnaturally sonorous, and may cause it to emit a hollow sound, when the lung is no longer permeable. We recollect a patient, in whom this was the case, though dissection after death, proved the left lung in its lower part to have been completely hepatized. It rarely happens, that the chest loses its sonorousness below a horizontal line passing through the nipples; for the larger bronchial ramifications remain pervious to the last; and when pus or serum is effused into the cavity of the chest, it necessarily accumulates in the inferior parts, extinguishing the hollow sound in these, while it is still emitted from the superior. Now as this fluid gravitates to the lowest parts of the cavity, it follows, that when the patient lies perfectly horizontal, it flows up along the spine, diminishing the hollowness of those parts, and increasing, or, to a certain degree, restoring that of the super-hypochondriac regions; a change which cannot occur if the lung be hepatized, and hence we are furnished with one means of diagnostic discrimination, between a disorganized lung and a pleuritic accumulation.

The sound acquires an unnatural intensity, when air, or some kind of gas, is extravasated or secreted into the cavity of the pleura (pneumo-thorax;) and in emphysema of the lungs, in which the air cells are permanently distended; but the most common cause, is extreme emaciation of the parieties; attended of course with absorption of the adipose matter about the base of the heart, and, perhaps also, of a portion of the cellular tissue of the pulmonary organ itself. We have seldom sounded the chest in cases of great atrophy, unaccompanied with organic legions of its viscera, without finding its sonorousness unusually intense and acute.

We shall conclude this branch of our inquiry with an extract from each of our authors.

The signs furnished by percussion are of great importance; we must not however depend on them always; it may happen that the sonorousness of the chest may be altered by causes foreign to the organs of the cavity. Thus every large tumor in the abdomen, pregnancy, ascites, diminish the sonorousness, and contract the thoracic cavity, thrusting up the lungs; but no cause, entirely independent of the pectoral organs, can produce a complete loss of sound.—Collin, pp. 21—22.

We must still admit, however, that the mothod of percussion is far from being complete, or generally available. It frequently affords no indication in phthisis; and in no case does it enable us to distinguish this disease from chronic peripneumony. Even in peripneumony it fails us in a great measure, when the inflammation is confined to the centre of the lung, or when both lungs are equally affected, and only in a slight degree. It does not enable us to distinguish the disease just mentioned from pleurisy, hydrothorax, or any other effusion into the cavity of the chest. It completely fails us, or rather certainly misleads us, in the disease called *Pneumo-Thorax.*

Laennec, p. 210.

Secondly, of Auscultation, and the practical use of the Stethoscope.

If the ear be pressed against any part of the chest of an individual, in whom that cavity and its contents are healthy, the entrance and exit of air in respiration can be distinctly heard. This species of auscultation or hearkening is denominated *immediate.* If instead of this, we interpose a solid and elastic body, between the thoracic parieties and the ear, the same sounds are heard; and this is denominated *mediate* auscultation, as being effected by the aid of means. This is the auscultation of Laennec; and the instrument which he invented for that purpose, is denominated the stethoscope, or chest inspector, as the Greek words of which it is composed may be taken to mean.

The annexed engraving, on type metal, and the description, are copied from the treatise of the inventor, and may aid those who are without his valuable work, in directing a common turner how to make the instrument.

Fig. 1. The Stethoscope or Cylinder, reduced to one third its actual dimensions.

*a.* The Stopper.

*b.* The lower half.

*c.* The upper half.

*d.* The auricular or upper extremity.

Fig. 2. Longitudinal section of the same, inverted.

*a.* The Stopper.

*b.* Point of union of the two parts.

*c.* The upper half.

Fig. 3. The same section, with the stopper removed.

Fig. 4. The Stopper.

*a.* The body of it, formed of the same wood as the rest of the instrument.

*b.* Small brass tube traversing the stopper, for fixing it in the tube of the Stethoscope.

Fig. 5. Upper half of the Stethoscope.

*a.* Body of it.

*b.* Screw (in the wood) for fixing the two portions together.

Fig. 6. Actual diameter of the Stethoscope.

*a.* Diameter of the canal of the Stethoscope.

The division of the instrument in the middle contributes nothing to its utility, and is designed merely to render it more portable; at the same time it is the most difficult part of the manufacture and the first to get out of order, so that in a new country where mechanics are scarce, it is better to keep the instrument permanently in one piece.

The wood of which it is formed ought to be of a medium density; it should be a foot long, an inch and a quarter in diameter, and its canal a fifth part of that space in calibre. A cylindrical form is the best, and from having this figure, Laennec frequently calls it the *cylinder*.

Fig. 1.

Fig. 2.

Fig. 3.

Fig. 4.

Fig. 5.

Fig. 6.

The following are rules useful to be observed in the management of the Stethoscope.

1. To procure stillness in the room in which the patient is placed, although to long practised auscultators, this precaution is in part unnecessary. Laennec repeatedly asserted that his organ of hearing was so adjusted to distinguish minute difference of sound, that he could at the same time, observe the movements of the heart—the noises produced during respiration by air, mucus, &c. in the lungs, and in the stomach and intestines; the noise of his own, and the patient's movements; and all this, while his students were moving and whispering round him.

2. The Stethoscope is to be used *without* the stopper, in exploring the phenomena of respiration, and the different *rattles;* and *with it*, to examine the circulation, and the voice.

3. It is not necessary that the part to which the Stethoscope is applied should be uncovered, but only that the covering be not too thick, be perfectly smooth, and not of a kind to produce a rustling noise, as silk or worsted will do.

4. The instrument must be applied perpendicularly, and so retained on the chest as to fit and press equally on every part, and at the same time the ear should be applied to the other extremity so as to exclude the external air, the hole of the cylinder being opposite the meatus auditorius.

5. In examining the respiration, the patient should be directed to respire a little more rapidly than natural, without creating a sound more audible than usual at a distance. In examining the voice, he may count aloud, slowly, in a natural tone of voice; and for exploring the different rattles, the patient should be directed to cough occasionally.

6. The observer should be careful not to place himself in a constrained position, and for this purpose it is often convenient to rest upon one knee.—pp. xiii, xiv.

To the foregoing judicious instructions for the use of the Stethoscope, compiled by the American Editor, we shall only add, that it should be held as we hold a pen in writing; and that when the patient is greatly emaciated, so that every part of the extremity of the instrument cannot be made to press equally upon the parieties, it is necessary to interpose a little lint, or cotton wool, or one of the ivory pieces used by Piorri in making percussion.

Our authors greatly prefer *mediate* to *immediate* auscultation; but the writer in the Medico-Chirurgical Review to whom this subject has been confided, thinks their preference not very well founded; and our own observations incline us to unite in his opinion. Even while composing this article, we have had occasion to examine a patient in whom the respiratory murmur was more audible and distinct, when heard by the application of the ear to the chest, than when listened to through the cylinder.

We are now prepared for studying the natural phenomena of respiration; without an accurate knowledge of which it is obviously impossible to understand the language of disease which is spoken through the stethescope.

On applying the cylinder to the thorax of a healthy man, we hear, at every inspiration and expiration, a gentle but very distinct noise, which indicates the penetration of the air into the pulmonary tissue, and its expulsion from it. It may also be perceived clearly that the air is received into a series of very small cavities, which expand to admit it, and not into one large extensive cavity.

This murmur is nearly equally strong in all points of the chest, but most so in those at which the lung is nearest to the surface of the skin, that is at the superior lateral and the posterior inferior parts. The hollow of the axilla, and the space between the clavicle and edge of the trapezius, are the points where it has most intensity.

Upon the trachea, larynx, and root of the lungs, the respiratory murmur is heard perfectly, but it has a particular character, which gives an idea of the air passing into a larger passage than that of the air cells. Neither do we distinguish the expanding of the pulmonary tissue, and the air seems to be attracted from the cylinder in inspiration and repelled in expiration. We may compare this mode of respiration, which we shall call tracheal, (bronchial) to the blast of a bellows.—pp. 28—29.

The student of auscultation, should, diligently and patiently, devote himself to the practical study of the phenomena described in the foregoing extract; for they must constitute his standard comparison in every clinical inquiry with the stethoscope.

The more frequent the respiration, the louder is the murmur; and hence, when it is obscure we should direct the patient to make short and quick inspirations. In women it is generally more audible than in men, except those of a feminine or nervous temperament. In children it is more intense than in adults; and among the latter, it is exceedingly different in different persons, all enjoying good health.

Having made himself acquainted with the healthy respiratory murmur, in its various modifications, arising from age, sex, temperament, and comparative corpulence, the student should next apply himself to the study of the voice.

On applying the cylinder upon the thorax, a confused resonance of the voice is heard; its intensity is not the same at all points.

The parts where it is strongest, at the axilla, the back between the inner edge of the scapula and the vertebral column, the anterior superior part of the chest, towards the angle formed by the junction of the sternum and clavicle.

At those spots the voice seems stronger and nearer to the observer than with the naked ear; in other parts, more particularly below and behind, it seems weaker and more distant, and produces a confused sound, in which nothing articulate can be distinguished.

In men who have a deep-toned voice, this resonance is stronger but flat, confused, and nearly equal at all points; it is clear and very distinct in persons whose voice has a sharp tone, in women and children. Finally, the agitated and trembling voice transmits the resonance very weakly, and in cases of aphonia is wholly absent.

pp. 31—32.

The student is now qualified for the study of pathological phenomena, or those modifications of the natural phenomena just described, which are produced by some kind of lesion of the lungs, or the parieties which enclose them. These phenomena are connected, 1. With the respiration; 2d. With the voice; 3d. With the respiration and the voice.

1. *Phenomena furnished by respiration.*

When the respiration becomes stronger than in the natural state, it assumes the character of that observed in infants, and has, for that reason, been named by M. Laennec, *puerile respiration.* This increased intensity of the respiratory murmur is never observed in cases of lesion of the lung, or of a part of the lung. It is met only in the healthy parts of the lung, the action of which is temporarily increased to supply the loss of the part diseased. Thus in pneumonia, it is not uncommon to find the respiration puerile in the parts not affected by the disease. However, we see this exaggeration of respiration coincide with a great dyspnœa, in some cases of asthma and of hysteric suffocation: it is difficult to account for this anomaly. We have thrice observed the respiration stronger than in the healthy state, in parts which have been attacked with pneumonia on the following day. This respiration was not truly puerile; it seemed to occur in a vast cavity immediately below the cylinder nearer to the surface of the body than it does in the thinnest persons.

In those three cases the peripneumony perhaps already occupied the centre of the organ, and the air vessels nearest to the surface alone were fit for carrying on respiration.

The respiratory murmur offering numerous varieties in the healthy state, it is only by examining different parts of the pulmonary organs that we can judge of its diminution. This comparison is always easy, for it is rare to find the respiration weakened in a whole lung, or in both together. The intensity of the noise offers many degrees, from a slight diminution to the most complete absence. The small extent of the movements of the thorax seems most commonly to be the cause of this diminution: it often depends also on the incomplete obstruction of the bronchial tubes of the middle size, owing to swelling of their lining membrane, or to the presence of accumulated sputa. We find it also in cases of membranes continuing soft, and only beginning to be organized.

The absence of the respiratory murmur may proceed from many causes. It occurs when the lung is become impermeable to the air, or when a liquid is interposed between it and the thoracic walls, or any other body accidentally developed, which prevents the transmission of the sound. It is rarely observed in the whole extent of one side of the thorax. The clavicles and the root of the lungs are the points where it occurs most rarely; perhaps never in the latter of these parts.

The tracheal respiration, of which we have spoken in treating of the natural phenomena, is sometimes observed in other points than those in which it is perceived in the healthy state. It cannot take place unless there be formed in the lung an excavation of considerable size, communicating freely with the bronchia, or being continuous with them. It has seemed to me also to arise from a hardening of the hepatized pulmonary tissue, which transmits to the ear the motion of the air in the great bronchial pipes, more noisy from the impossibility which the induration offers to the penetration of the air into the bronchial vesicles. Whatever be the intensity of the respiratory murmur, it may be either pure, which indicates that the bronchia are perfectly free, or it may be attended with rattles.

pp. 38—41.

The last word of this extended extract, introduces us to several pathological phenomena deserving the strictest attention. The term *rale*, employed by Laennec and the different writers of his school, and translated *rattles*, includes 'every kind of noise attending the circulation of air in the bronchia and vesicles, different from the murmur produced by it in the healthy state.' The *rattles* are seldom heard over the whole extent of the chest. They are, in the majority of cases, perceptible but in a spot not very extensive, the respiratory murmur remaining natural, or becoming puerile, that is excessively loud, in other parts of the chest. 'The rattles,' as Collin observes, 'announce either the contraction of some part of the bronchial tubes, or their being gorged by some fluid, or, finally, a similar state of the air vesicles.' It is obvious enough, that the noise denominated *wheezing* is included under this definition, along with that to which the word *rattles* is usually applied.

Collin, systematysing the observations of Laennec, enumerates four kinds of rattles ; the *dry sonorous*, the *hissing*, the *mucous*, and the *crepitating*. Some of them are best heard when the patient coughs; others, and the greater number, in connexion with the respiration; they may exist singly, or coetaneously in different parts of the chest; be permanent through the whole course of the disease, or intermit. Regarding every thing relative to this genus of morbid phenomena, as of deep and curious interest to the clinical practitioner, we shall transcribe, entire, the condensed and instructive remarks of our author on each species.

*The sonorous rattle.*—This consists in a sound more or less grave, and often extremely noisy, resembling at one time the snoring of a sleeping man, at another the sound produced by rubbing the string of a bass vial with the finger, and frequently the cooing of a dove. It appears to be owing to the contraction of the bronchial tubes by the swelling of their lining membrane, or to some change in the form of these canals, perhaps to the thickening of the spurseperons at the point of division of the bronchia, a thickening we almost constantly observe in subjects who have fallen during the continuance of a catarrh, either acute or chronic.

*The sibilant or hissing rattle.*—It resembles a prolonged hiss, and accompanies either the end or commencement of inspiration, or of expiration. It is grave or acute, dull or sonorous. These two varieties are sometimes met together in different parts of the lung, or succeed each other in the same point, at closer or more distant intervals. It depends on the presence of mucosity, not very abundant, but thin, viscous, obstructing more or less completely the small bronchial ramifications which the air must traverse to reach the air vesicles. It seems to me to indicate an alteration of the lung, deeper than the preceding, that is to say, occupying finer ramifications; and when it is heard in a great part of the organ it is attended with more constraint of respiration. It is during the existence of the sibilant rattle that we observe those mucous sputa of an orborescent appearance, presenting to the eye the form, calibre, and ramifications of the small tubes from which they have been propelled by the effort of coughing.

*The mucous rattle.*—This rattle, produced by the passage of the air through sputa accumulated in the bronchia or trachea, or through softer tuberculous matter, denotes by its nature the unctuous untenacious state of the liquid which fills the air tubes. At one time it is weak, and produced at remote intervals; at another it is strong and persistent. In the first case it may be judged that the column of air meets only at intervals the mucosity which produces it; in the second, that the bronchia are almost entirely filled with it. Carried to the highest degree it constitutes gurgling, the name by which we characterize the noisy murmur caused by the agitation of tuberculous matter, or purulent sputa, with the air traversing them.

*Crepitating rattle.*—This consists in a noise which may be justly compared to that of butter boiling, or of salt crackling on a hot bassine plate, or to that produced by squeezing a bit of dried healthy lung between the fingers. It seems to be owing to the exhalation of blood into the air cells, observed in a lung affected with the first degree of pneumonia, of which the crepitating rattle is the pathognomonic sign. A new proof of the reality of this cause may be drawn from an analogous kind of injury in œdema of the lung, characterised by a variety of this rattle of which I am about to make mention. This variety has very striking characters; and if we have not made a particular species of it, it is because it is difficult to describe its differences exactly, though they are very sensible to the ear; and it is enough to have heard the two kinds once, not to confound them afterwards. The name of subcrepitating, given to it by M. Laennec, indicates its character well. In reality, this rattle is analogous enough to the first, but it gives a sensation to the ear that the liquid which produces it has more tenuity and less plasticity than the crepitating rattle. On opening the dead bodies of subjects affected with œdema of the lungs, we find this viscus filled with a limpid serous fluid without viscosity which fills up the bronchia and vesicles, and is filtrated into the interlobular cellular tissue, rendering the lung flaccid and inelastic.

Such are the different rattles audible with the cylinder. We see by their description that they cannot be mistaken, and each offers very striking characters; but their differences are often less sensible, and shades, which practice teaches us to seize, and which words cannot express, establish a sort of transition between each of them, and indicate a mixed lesion, more or less like that indicated by each individually.—pp. 43—47.

2. *Phenomena furnished by the voice.*—These are embraced under three heads—*resonance*, *pectoriloquy*, and *ægophony*. We shall consider them separately.

*Resonance.*—We have found not a little difficulty in distinguishing between resonance and pectoriloquism, in the earlier periods of our auscultic trials; and the translator of Collin, cautions beginners against this mistake. Resonance, in the language of auscultation, is a sonorous resounding of the voice, in a part where it cannot be heard in health. But it is not the speech which is heard. The observer perceives no articulate sounds. A confused noise only, reaches his ear through the cylinder.

*Pectoriloquy.*—This word explains itself. It is the speech of the breast. The articulated voice of the patient seems to pass from the spot upon which the stethoscope rests, along or through the tube of that instrument, to the ear of the observer. The sound is similar to that heard when the cylinder is applied to the trachea, and by thus applying it to a person in health, the auscultic pupil will soon become familiar with the sound which characterizes the morbid phenomenon under consideration.

This phenomenon is always owing to the presence of excavations in the lung, communicating freely with the bronchia, and wholly or partly empty. It may be met with in all points of the chest; but the parts in which it is observed the most frequently, are, below the clavicles, in the hollow of the axilla, the space between the clavicle and the trapezius muscle, the supra and infra spinal fossa. These parts all correspond to the summit of the organ, and it is in that part in reality, that excavations, produced by the softening of tubercles, are most frequently detected.

Pectoriloquy offers some variety, arising from difference in the tone of the voice, the size of the cavity, its form, the firmness or softness of its walls, their adhesion or want of adhesion to the costal pleura, or to the difficulty with which the air penetrates them.

1. The more acute the voice is, the more evident is the pectoriloquy; it is almost always imperfect and sometimes doubtful in persons of a deep-toned voice. Aphonia does not make it disappear completely; and it often happens in these cases, that we can distinguish what the patient says, better with the cylinder applied to the point of the chest where the cavity exists, than with the naked ear at the same distance.

2. Perfect pectoriloquy requires the cavity to be of moderate extent. In very large cavities it changes to a stronger, deeper sound, analogous to that of the voice transmitted to some distance through a scroll or trumpet made of paper. In very small caverns it is often doubtful, particularly when the excavation is central, and surrounded with parts of the lung still permeable to the air.

3. The winding disposition of the cavities, or the direct communication of a number of excavations with one another, give something of a confused or stifled sound to the pectoriloquy; the voice seems badly articulated.

4. The firmer and thinner the walls, the more perfect the pectoriloquy. When cicatrization has produced a membrane of a fibro cartilaginous nature over the whole surface of one of the cavities, pectoriloquy acquires a metallic tone, sometimes so noisy as to injure the clearness of the perception of the sounds.

5. An excavation placed near the surface of the lung, the thin wall of which does not adhere to the costal pleura and skins in expiration, does not yield pectoriloquy; we then recognize its existence by other characters. On the other hand, a superficial excavation with thin adherent walls, gives a striking pectoriloquy with a force which fatigues the ear.

6. The less fluid there is in the excavation, the more evident is the pectoriloquy, because the communication with the bronchia is then usually large, and permits a free access to the air. However, this communication may be destroyed more or less completely, and for a longer or shorter time, by the stagnation of the sputa in the bronchial tubes; it is this which sometimes renders pectoriloquy doubtful, and gives it that intermittent character not rarely observed. There are days in which we can hardly find one case of it in a ward in which the day before we observed a great many: we perceive then that in most cases the expectoration has been but small, or wholly deficient.—Collin, pp. 50 53.

As pectoriloquism is produced by the voice resounding in pulmonary excavations, it may be regarded as the pathognomonic sign of the existence of accidental cavities in the lung; nevertheless, Collins and Cruveilheir have met with cases which seem to show, that pectoriloquism may be emitted by the larger bronchial tubes, when the lung, from chronic pneumony or some other cause, has become hepatized or indurated, so as to transmit the vibrations of the voice more perfectly. This kind of pectoriloquism is obviously allied to resonance. To this phenomenon, Laennec has given the name of *bronchophony.* We have frequently met with it in lean persons; in some of whom it seemed to be a natural sound, rather than the product of disease.

*Ægophony.* This is the hægophony of the first edition of Laennec; who bestowed upon it this name, from its resemblance to the bleating of a goat. It has been confounded with pectoriloquy, and still oftener with bronchophony.

Ægophony is a strong resonance of the voice, more sharp, more acute, than that of the patient; in some degree argentine, jerking, and tremulous, like that of a kid. This phenomenon may be produced in the whole extent of the chest, on one side only, or on both sides at once; but it is rarely that it is not confined to a tolerably circumscribed space, of which the vertebral column, the inner border of the scapula, its inferior angle, and its outer edge, form the limits.

When it exists on both sides together, it is difficult to decide whether it be a consequence of disease: in some subjects the natural resonance of the voice at the root of the lung has this sharp bleating character.

Hægophony varies much in force and extent; but however weak it may be, it seems to me always to indicate with certainty the existance of a moderate quantity of liquid in the cavity of the pleura, or of pseudo membranes tolerably thick, and still soft.

When the effusion becomes too abundant or too small in quantity, hægophony ceases to exist. I have never found it when the effusion of the liquid took place very rapidly, and the affected side was filled almost as suddenly.—pp. 56—57.

Of the various morbid phenomena of the voice, this has always seemed to us the most difficult to explain. The following are the observations of Collin, touching this point, from which the reader will perceive, that a true explanation is, perhaps, still a desideratum.

Can this phenomenon be explained by the quivering of the voice on the surface of a liquid, as M. Laennec formerly thought? Or is it owing to the flattening of the bronchial tubes, as he teaches at present?

A woman presented hægophony towards the root of each lung in turn: she died. No liquid was found extravasated; the flattening of the bronchia was not very evident.

Another died after long suffering. For three years she had been pectoriloquous in the summit of the lung, and hægophonous at the root of that organ in a very circumscribed space. There was no extravasation, and although surrounded by a very dense pulmonary tissue, the bronchia did not appear to me altered in their form.

A man was carried off by pneumonia; the dullness of percussion, the presence of hægophony, and the absence of all respiratory murmur, had made the existence of effusion be suspected: four hours before death the patient was still hægophonous. There was no liquid extravasated however; but there was a tolerably thick and slightly consistent layer of false membrane upon the root of the lung. The bronchial tubes offered nothing particular. I will confess that it is very difficult to discern whether there is flattening of these tubes or not; they are not naturally cylindrical, and perhaps a very slight degree of closing may cause hægophony.

These three facts, unique as far as I know, do not hinder me from believing that this phenomenon indicates with certainty a moderate extravasation of fluid into the cavity of the thorax; but they seem to me sufficient to make us reject the first explanation of hægophony, and insufficient to prove the second.—pp. 57, 59.

3. We come in the third and last place, to notice certain morbid phenomena connected both with the respiration and the voice. They are *metallic respiration*, *metallic resonance*, and *metallic tinkling*. Several different lesions give rise to the sounds which are expressed by these terms.

These morbid states are a fistulous communication between the cavity of the pleura and the bronchia, and the accumulation of a certain quantity of air in the sac formed by that membrane; an effusion both liquid and gaseous, with or without communication; finally, a very large excavation, with thin and compact adherent walls. Metallic respiration, and metallic resonance, will exist in the first case; metallic tinkling will be joined to them in the third, or will be found alone if there be no bronchial fistula.

If we make a patient in whom this communication exists, breathe strongly, the air in penetrating the pleural cavity, produces a murmur similar to that caused by blowing into a rather narrow-mouthed metal vessel. If we make him speak, his voice bounds under the cylinder, and resounds as if he spoke in a cistern. This is the more striking, as the phenomenon is sometimes evident only at the end of the sentence, and seems to be an echo.

Finally, if an effusion both gaseous and liquid exist, and we make the patient rise, to examine him, it sometimes happens that we hear a sound of short duration, similar to that of a drop of water falling into a decanter three-fourths empty. It seems as if a drop remained sticking to the upper part of that cavity, and falling into the lower part, then occupied by the effused fluid, produced this noise by its fall into the mass of liquid.

These phenomena never failed to become evident, from time to time, at least during the continuance of the symptoms they denote. It is scarcely necessary to say that we often examine the patient several times before an opportunity occurs of hearing them. The obstruction of the fistula or bronchia communicating with it makes them disappear. A certain proportion between the liquid and gaseous effusion is necessary for their production in a very distinct degree.

Agitation of the air, its passage through a narrow opening, and the resounding of the voice in a large cavity with firm walls, half solid and capable of vibrating with force, easily account for the metallic respiration and resonance. The explanation of the tinkling which I have given, seems probable enough.—pp. 61, 64.

On the subject of the metallic tinkling, Laennec, in the last edition of his book states, that 'it always originates in a morbid excavation within the chest, containing both a liquid and a gas.'

We have now travelled through the *elements* of pulmonary auscultation. In studying them, the greatest difficulties seem to us to be with the voice and speech; but even these are far from insuperable. They may be vanquished by any one who unites to an accurate ear, a tolerable degree of professional enterprise, judiciously directed. Having this conviction, we are sorry to be obliged to say, that most of the physicians of the United States remain, nearly, ignorant of the whole subject. In this city, where there are between thirty and forty practitioners, we are not aware, that more than two or three, are at all acquainted with the use of the stethoscope, and even doubt whether a majority of them have studied the theory of auscultation.

The close of the first part of Collin's manuel, consists of two short articles on the *mensuration* and *succussion* of the chest. The object of mensuration, is to ascertain the dilatation or contraction of one side of the chest. A piece of tape, or something flat and inelastic, should be passed round from the spinous processes to the centre of the sternum. Dilatation of the chest, is the consequence of serous or purulent effusion into the cavity of the pleura. Contraction, in the opinion of Laennec and Collin, is always the consequence of pleurisy. The following is the explanation offered by the former in his treatise on diseases of the chest:

This morbid contraction of one of the thoracic cavities arises from a somewhat irregular termination of chronic pleurisy, or of the acute pleurisy become chronic. In these cases the sero-perulent effusion having continued for a long time, the false membranes which invest the pleura and lungs acquire a particular hardness, and an incipient organization, which render them incapable of being converted into cellular substance. When the effusion is absorbed, the lung long compressed by it, and further bound down by a strong false membrane completely investing it, cannot dilate itself sufficiently promptly to keep pace with the progress of the absorption: the ribs, consequently, contract, and the cavity of the chest is thus diminished. When the fluids are completely absorbed, the costal and pulmonary exudations come into close contact and finally unite, so as to form only one substance. The consistence of this becomes daily firmer, and, after a few months, acquires the consistence and all the other characters of a fibrous or fibro-cartilaginous membrane.—Laennec, p. 132.

By succussion, we can frequently detect the existence of a fluid in the sack of the pleura. The chest must receive 'one or more rapid abrupt shakes,' which need not be very violent. The sound is quite similar to that of shaking a bottle partly filled with water. In the opinion of Collin, there must be both gaseous and liquid effusions, in certain proportions, or succussion will afford no satisfactory result.

We come now to inquire into the practical application of the stethoscope in the diagnosis of pulmonary diseases, and pursuing the order already adopted, shall begin with those in which a morbid state of the respiration affords the principal indication. Our remarks, however, will not be limited to auscultation, although our great object is to ascertain and illustrate its value. Percussion, succussion, mensuration, and an examination of the thoracic movements, are auxiliary means of diagnosis; and in the opinion of Collin, their relative aid to auscultation, is generally in the order in which they are here enumerated.

1. *Pleurodynia.*—-False pleurisy. Thoracic rheumatism? 'Percussion returns a dull sound,' and the 'respiratory murmur is weak in a greater or less extent, or wholly absent.'

2. *Bronchitis.*—Pulmonary catarrh. Percussion, mensuration, and succussion give but little information; it is far different with the stethoscope.

The signs it furnishes are truly pathognomic; they vary as the catarrh is dry or moist. In the dry catarrh we observe weakness, or even absence in the respiratory murmur in parts of the lung of greater or less extent; but these change every moment, and during the course of a short examination, may occupy different points in turn, so that the murmur may become distinct where it was absent, and absent where it had just before been clearly perceived.

This weakness of the respiratory murmur is very often accompanied with the dry, sonorous, or the sibilant rattles. The first, little variable; the second, very much so, disappearing for a longer or shorter time after the effort of coughing, or even without any evident cause; returning abruptly, assuming an increased intensity, or losing that which it had at first. Sometimes, however, both are constant, intense, and occupying the greatest part of the organ: the catarrh is then extensive and violent.

In the humid catarrh the same phenomena may exist, but they are then usually attended with a third, the mucous rattle; or this alone is heard, and is sufficient to characterize the complaint. Less frequently varying its situation than the hissing rattle, the mucous rattle presents shades, either in force, frequency, or extent, which make known the different degrees of the catarrhal affection. Catarrh may be easily confounded with emphysema of the lung and pulmonary phthisis.—Collin, pp. 88, 89.

In croup, a resort to the stethoscope may enable us to decide whether the inflammation has extended from the larynx to the bronchia and air cells.

3. *Hæmoptysis.*—Pulmonary Apoplexy. In this malady, the chest during the first stage remains as sonorous as before the attack. Examined with the stethoscope, the respiratory murmur is altered.

The crepitating rattle develops itself in more or less numerous and circumscribed points of the lung. The spaces between these still present a perfect, and even puerile respiratory murmur.

At the end of a longer or shorter time it ceases to be heard; an abundant mucous rattle in large bubbles, succeeds to it, indicating a copious exhalation of blood into the air cells and bronchia, occupying very soon the whole lobe or affected lung, and the bloody expectoration soon confirms the diagnostic already pointed out by those phenomena.— pp. 90, 91.

4. *Œdema of the lungs.*—Anasarca pulmonum. It generally affects both lungs. Under percussion, the sound is natural or duller.

Respiratory murmur scarcely distinct, marked in almost the whole viscus, but chiefly in the back and inferior parts, by a sub-crepitating rattle, slight and energetic, but constant in its existence; the respiration sometimes puerile in a small extent of the upper part of the organ. Such are the symptoms of œdema of the lung.—pp. 91, 92.

5. *Emphysema of the lungs.* Sonorousness from percussion excessive in certain parts of the chest.

The murmur of respiration is very weak or wholly absent in all the points attacked by emphysema; a slight sibilant rattle similar to the clicking of a small valve, or a sonorous rattle imitating the cooing of a dove, is heard in deep inspirations, and sometimes also in expirations. The contrast of this greater resonance (or hollowness) of the thorax, with the diminution or absence of the murmur, forms a characteristic symptom of this disease. It is true that these characters of the respiration and the existence of the rattle are inconstant and variable; but they always remain a long time, and their changes are only momentaneous.

When the complaint is chronic and very extensive, another sign drawn from the mensuration may be added to those just enumerated, the dilatation of the side affected; and if the affection is on both sides, the almost cylindrical form of the chest projecting behind and before. Emphysema, sometimes distinguished with difficulty from pulmonary catarrh, may also be taken for a pneumothorax without liquid effusion.

Let us first explain how it differs from catarrh. In catarrh the suspension of the respiratory murmur is of short continuance in the same point; its return is sometimes marked by a strong and even puerile respiratory murmur; a frequent rattle attends it.

In emphysema, the suspension of respiration at the same point is longer, sometimes even permanent: when it ceases, the sound always remains more feeble, particularly if the complaint be ancient. The hissing rattle is rare, and badly characterized; the sonorous rattle, imitating the cooing of the dove, is constant, and almost never determined by a simple catarrh.

Besides, in this last affection, the movement of the sides is free; the respiration presents no constant inequality; the chest preserves its natural capacity and hollow sound. In emphysema, one side is often less moveable than the other; inspiration is always very short, relative to expiration; the chest dilates, and acquires a tympanitic resonance.

It is scarcely of use to say that percussion establishes at once the difference between emphysema and the other diseases of the chest in which respiration appears to the cylinder more feeble or absent: I only except pneumo-thorax.—pp. 93,—95.

6. *Pneumonia.*—Inflammation of the pulmonary tissue. In this very momentous affection, the stethoscope supplies us with much important information. In its several stages, the phenomena revealed by the cylinder, are different.

The respiratory murmur is feeble, in all parts where the sonorousness is diminished scarcely distinct, or sometimes covered by a crepitating rattle; at one time dull, at another sonorous enough, and the presence of which indicates both the nature of the alteration and the whole extent it occupies. The respiration then often becomes puerile in the other lung, and in all the parts of the effected lung yet remaining healthy.

These phenomena very soon change. If the disease terminates by resolution, the crepitating rattle diminishes in intensity every day; the murmur of respiration approaches more and more to the natural state; the movements of the chest resume their rhythm, their extent, and simultaneousness; the sound returns, and the mucous rattle, in a greater or less degree, indicates the change of expectoration.

On the contrary, if the lung passes to the state of hepatization, the alteration of the movements of the thorax continue, the sound becomes completely dull; the crepitating rattle ceases, but the respiratory murmur does not return; the smallest quantity of air cannot penetrate the hardened tissue of the lung. Respiration is wholly absent, or if heard, is so only in the vicinity of the large bronchial tubes; it is then tracheal, cavernous, and often very loud; the hollowness of the voice redoubles in all the affected parts; often in induration of the upper lobe even a true pectoriloquy begins to complicate the diagnosis, and throw doubts upon the nature of the affection. We must have recourse to the commemorative circumstances, to the general symptoms, to prevent our supposing the existence of pulmonary phthisis.

When the disease is of small extent, nature and art exert their powers, and are often at this period crowned with success; the disease retracing its steps by the same way it advanced, presents in turns and in inverse order the phenomena before observed. But if the complaint continue its progress—if the suppurative process seizes upon the pulmonary tissue, the movements of the chest become smaller and smaller, feeble and more difficult; to the first causes of their alteration general debility is added. The sound remains dull; a large bubble mucous rattle is first developed in insolated points, then in all the morbid part. It soon degenerates to the gurgling rattle; the pus collected in an abscess bursts into the surrounding bronchia; a communication is formed between these tubes and this accidental cavity, and pectoriloquy manifests itself, at first obscure, whatever may be the point the disease occupies.

We see from this abridged sketch, that each stage has very striking characters; and that if we have been called in at the commencement of the complaint, and have been able to follow the progress of the disease, step by step, it is easy to predict, in case of death, the extent and degree of lesion that will present itself. It is not so if we see the patient for the first time in the second state, when the lung is hepatized. In fact some ribs are immoveable, the sound is dull, the respiration absent; but those symptoms are common to empyema and hydro-thorax. Here the five modes of inquiry are insufficient, and we must seek for information in the amnestic signs and progress of the disease. Percussion and auscultation could not prevent an error always disagreeable, sometimes fatal.

In the third period, that of suppuration, it is difficult to guard against a mistake, less unpleasant indeed, but which may compromise the reputation of the physician.

The cavernous respiration, the gurgling, and pectoriloquy exist, and the general symptoms are nearly those of pulmonary phthisis.

As to the chronic pneumonia, after a vomica has formed and burst into the bronchia, we must apply to it what we have said of the second degree, that of hepatization, of the third, that of suppuration.

It remains to say what signs distinguish pneumonia from pleurodynia, from the first degree of pulmonary apoplexy, and from œdema of the lung. The crepitating rattle in the first state; the dull sound on percussion and perfect absence of respiratory murmur in the second; the dullness, mucous rattle, and pectoriloquy in the third stage, distinguish pneumonia from pleurodynia. In most cases percussion would prevent our confounding this disease with pulmonary apoplexy, if the examination of the movements of the chest did not already afford a good diagnostic difference. In fact, in pulmonary apoplexy the respiration is always complete; it is usually incomplete in pneumonia. The sound is always more or less obscure, often wholly absent, in the first stage of pneumonia when the crepitating rattle exists; it remains clear in the first stage of pulmonary apoplexy. The crepitating rattle is seldom widely spread in pneumonia; it is so usually in apoplexy. The mucous rattle suddenly succeeds the crepitating in the latter.

In pneumonia the absence of all respiratory murmur exists sometime between the moment in which the crepitating rattle ceases, and that in which the mucous commences.

It is the same with œdema of the lung.—pp. 96, 101.

We shall not insult our readers by an apology for this lengthened and most interesting extract. Pneumonia is not only a common and fatal, but too often a most insidious disease; and every thing which can throw light upon its diagnosis deserves the greatest attention.

7. Pleurisy. Empyema. Hydro-Thorax.—Some of our readers may perhaps, not see the propriety of uniting these affections into one article. This will be the case, with all those, (and the number is not small,) who are unapprized, that in every case of pleurisy, there is almost from the beginning, a serious effusion into the cavity of the inflamed pleura. In most of our current works on pathology and the practice, but little is said on this topic; the effusion which they recognize, being that of fibrin or coagulable lymph, agglutinating the pleura pulmonum with the pleura costalis. The researches of Lænnec on this subject constitute, in the language of his able translator, Dr. Forbes, an admirable piece of pathology. We earnestly recommend it to every reader, who would understand the morbid anatomy of the pleura. It is on the effusion to which we have referred, that the similarity of pleurisy, empyema and hydrothorax principally rests.

In the diagnosis of pleurisy, the stethoscope affords information which is truly pathognomonic; but other signs must be studied at the same time; and to enable such of our readers as may not possess the work of either Lænnec or Collin, to employ the stethoscope, successfully, in the diagnosis of this malady, we shall lay before them the whole that is said on this topic by the latter.

The signs afforded by the different modes in this disease, vary according to their being considered at the commencement, or after effusion has taken place.

In the commencement, that is before a serous or plastic liquid has been accumulated between the pleura and the lung, the movements of the thorax are enfeebled or almost wanting on the affected side. We have daily opportunities of observing that the ribs of the affected side alone are immoveable, while the others continue to move. Respiration is frequent, particularly if both sides are affected at once, quick in inspiration, interrupted, irregular. These characters continue during the whole acute stage of the complaint.

Percussion is painful, but gives the same results as in the sound state.

The respiratory murmur is enfeebled, but pure, if the disease be not complicated; the capacity of the chest is not augmented. Finally, the symptoms are as in pleurodine—a disease it is impossible to distinguish from the commencement of pleurisy, except by the general symptoms.

When the effusion has taken place, and is in small quantity, the resonance usually becomes obscure in the lateral and posterior inferior parts, or in any point of the thorax, tf the disease be circumscribed and an anterior pleurisy has produced adhesions sufficient to confine the liquid.

The cylinder applied along the spinal edge of the scapula, towards its point or its outer edge, or in fine in any other place, even under the clavicles, according to the extent of the effusion, or the point it occupies, renders evident that sharp, tremulous, jerking voice, called by M. Laennec hægophony. The respiratory murmur is absent or scarcely distinct, in all that part in which the sonorousness is altered. It becomes sometimes puerile in the upper parts of the lung.

If the effusion is very considerable in the beginning, or becomes so in the progress of the affection, the sound becomes wholly flat, the hægophony disappears, the respiratory murmur is no longer audible, unless short adhesions retain parts of the lungs near the ribs, and prevent them feom being thrust back. The intercostal spaces widen, rise to the level of the ribs: these become flattened; the affected side enlarges, it becomes unfit for respiration, and its immoveability contrasts with the greater mobility of the opposite one on which side the respiration acquires the puerile character.

If absorption of the liquid takes place, hægophony reappears when the quantity is reduced to that necessary to the production of the phenomena: it then gradually diminishes as the quantity lessens, and finally disappears altogether when the absorption is completed. However, the sound still remains a long time flat, and the respiration absent or feeble; the ribs fall, the intercostal spaces sink, are effaced; the chest contracts, and that side never assumes either its former volume or mobility.

The resonance increases, and the respiratory murmur is heard with any force only when the pseudo-membranes have been converted into an organized tissue similar to cellular, membrane, fibro-cartilage or bone.

No disease can be confounded with pleurisy, so long as hægophony exists, except commencing hydro-thorax.

This phenomenon is always a pathognomonic sign of these two affections; the other local and general symptoms serve to distinguish them.

But when the effusion is copious and the disease chronic, if we have not attended to its progress, we may take pleurisy for a hydro-thorax or chronic pneumonia, and reciprocally these affections for a pleurisy.

The anamnestic signs and the general symptoms alone can establish the distinction; this is the more important, as little can be done for chronic pleurisy, while powerful and efficacious remedies remain for hydro-thorax and pneumonia. The operation for empyema is the only relief in chronic pleurisy, and would have more success without doubt if earlier performed.

However, the dilitation of the thorax, the perfect immobility of the ribs, seem to me not to exist in chronic pneumonia, and establish constant differences of character, unless the diseased side, having been before affected with contraction, has been incapable of enlarging.

As to the possibility of confounding chronic pleurisy and pulmonary phthisis, I think that even when there is not pectoriloquy, there are other characters sufficiently distinguishing to render this mistake hereafter impossible (*avoidable*, Ed.;) and even when they are complicated, it is often easy to distinguish them from each other.

The difference between pleurisy and pleurodyne is easily laid down. In pleurisy, when we observe the respiration incomplete, the resonance obscure, and the murmur absent or feeble, there will be hægophony at the same time; this phenomenon never exists in pleurodynia. If the effusion was copious enough to destroy the hægophony, there would be dilatation. Moreover, the error in any case cannot exist long.—101, 106.

Pneumo Thorax.—In this affection percussion raises a hollow tympanitic sound, while the Stethoscope detects no respiratory murmur, except near the root of the lung and there it is feeble. Thus the union of the two methods enables us to say that the case is not emphysema, in which the sound under percussion is not unnatural in degree, and the respiratory murmur scarcely ever entirely gone. The same union serves equally to distinguish this complaint from empyema and hydrothorax, for although the respiratory murmur is absent in all, the hollow sound under percussion is wanting in the latter. When there is a fistulous opening from the lung into the cavity of the pleura, there will be metallic respiration and metallic resonance. If the effusion is both liquid and gaseous, and a fistulous opening has been established, metallic tinkling will be superadded. In this case, moreover, percussion will afford no sound, if made exterior to the part in which the liquid is accumulated, while the sonorousness will be great in the parts above, which have the gaseous accumulation; and as these two fluids may change places, from an altered posture of the patient's body, it happens, that the same part of the chest at different times will sound differently under percussion.

9. Phthisis Pulmonalis. Tubercular consumption. With the people, and not a few of their medical advisers, every chronic disease attended with dyspnœa pain, cough, expectoration, and fever, is a consumption. But a great variety of læsions of the pulmonary organ may give rise to these symptoms. The principal are bronchitis, peripneumony, pleurisy, and phthisis—the anatomical characters of which are essentially different, whatever identity superficial observation may admit in their symptoms. To the study of this morbid anatomy, Lænnec devoted himself with a degree of success, that must render his work a durable monúment of genius and industry. To have made dissections, and published descriptions the most accurate, would have been of little value, unless a connection had been established between the symptoms during life, and the morbid appearances after death. Of this important truth, so little regarded by Lieutaud and many other writers on morbid anatomy, our author was so well aware, that its influence is impressed on every page of his admirable work.

So long as the symptoms just enumerated, and these only, were placed by the anatomical pathologists, in connexion with the læsions of structure revealed by dissections, all was confusion and uncertainty. Those symptoms, being common to most pulmonary disorders, have, indeed, a generic character, and are almost equally connected with every species of pulmonic derangement. It was reserved for Lænnec to institute a new system of semeiology, and to show that each læsion is indicated by special signs. Thus the mucous rattle is in some degree pathognomonic of bronchitis, and ægophonism of pleurisy. Knowing this, we are led to ask whether the same means of diagnosis can be made to establish the existence of tubercular phthisis? We answer yes,—but not while the disease is yet, in its early stages. When a tubercle, or cluster of tubercles, has softened and been expectorated, a cavity is left, for there is no reproduction of the pulmonary tissue. In this cavity we perceive by the aid of the stethoscope, that resonance of the articulated voice of the patient, to which the name pectoriloquy is applied; and hence that phenomenon is pathognomic of tubercular, or, in common parlance, genuine consumption.

To understand this subject properly, the student should read with attention the two chapters of Læennec which treat of pulmonary phthisis; meanwhile we shall present him with a condensed account by Collin, recommending it to the serious attention of every practitioner who is ambitious to answer correctly the oft repeated and momentous question—is this a case of true and incurable consumption?

To lay down the semeiology of pulmonary phthisis the more clearly, we shall admit it to have three stages, although this complaint is seldom constant in its duration, and so obscure in its progress as rarely to favor this division.

The first of those periods, that in which an inconsiderable number of tubercles are developed in the lung, presents on examining the local phenomena and the general symptoms, only the appearance of a catarrh of greater or less severity; it is sometimes concealed from observation and does not seem to exist.

In the second stage, the tubercles are already in sufficient numbers to stifle, as we may say, the tissue of the organ in those places where they are most frequently observed to accumulate and give rise to phenomena, insufficient to enable us to say with certainty that the disease exists, but enough to make us suspect it.

Finally, in the third, the melting, the softening, and evacuation of the tubercles, gives place to a phenomenon which is always a certain sign of this affection and the shades of which point out its extent and its intensity.

The change in the movement of the thorax are extremely variable in this tedious and melancholy complaint. They may be all met with during its progress, but are never of great use in the diagnosis.

In the second stage, the upper part of one side of the chest frequently returns a flatter and more obscure sound than natural, on percussion. The Stethoscope applied to this spot makes known a feebleness, or even complete absence of the respiratory murmur, generally in an extent rather limited; the voice resounds with more force under the instrument; but these symptoms only become signs of the complaint when they exist on one side alone, and are constant; it is only the comparison between the healthy and the diseased side which shows their value.

The sound on percussion very soon returns, and sometimes acquires even more intensity, or it loses still more of its distinctness, and from being obscure as before, becomes quite dull.

Pectoriloquy becomes at first doubtful, but does not delay long to acquire its greatest perfection, and ends by being again only imperfect, if the disease, continuing its progress, produces vast excavations. The phenomena produced by the catarrh are extended and aggravated from day to day, and continue to the moment of death.

If in all cases those two very striking periods, and this succession of phenomena existed, phthisis would cease to be a disease so often difficult to recognize; but how frequently does it not happen, that patients fall before the softening and evacuation of the tubercles, even before their accumulation has altered the sonorousness of the chest, or injured the perfection of respiration.

The melancholy information acquired by the cylinder is certainly precious; but in most cases the disease is beyond the reach of art when it is discovered.

Chronic pulmonary catarrh must then be always confounded with phthisis, so long as pectoriliquy, or the three phenomena mentioned as signs of the accumulation of tubercles, do not exist.*

Phthisis will also be confounded with acute or chronic pneumonia, occupying the upper lobe of the lung; the distinguishing charac-

* The chief diagnostic symptom is the rattle which attends the catarrh.–Transl.

ters can only be found in the general symptoms and the appearance of the expectoration; and these are but little to be trusted to.

It will more rarely resemble emphysema of the lung, and percussion and the general symtoms will easily distinguish them.

The dilatation of the bronchia, a common consequence of long-continued pulmonary catarrh, gives rise also to the phenomena of pectoriloquy. It is then impossible to avoid a mistake; time alone, and the progress of the disease, sometimes deceive us.—pp. 106, 110.

With this extract we close our present notice of the Stethoscope in diseases of the lungs; in our next number we shall enquire into its nature as an instrument of diagnosis in maladies of the heart.

Convinced as we are of its importance, we cannot refrain from urging upon such of our readers, by far the greater number, as have not yet given it a trial, the *duty* of doing so. It is an undeniable fact, that we every day see patients struggling through the successive stages of mortal diseases of the thoracic viscera, and consigned to the grave, without our knowing or being able to say more, than that they have a *decline*, or a complaint of the heart. We even venture to assert, that many a patient has been suffered to die of pulmonary disease, without having been subjected either to thoracic pressure, succussion, admeasurement, percussion, or the stethoscope—omissions not less prejudicial to the sick, than derogatory to the character of the profession. Let not the difficulties attendant on these clinical processes, deter any one from attempting to practise them. None of them require more time and study,than many other operations,for the performance of which the most unambitious member of the profession, would be ashamed to remain unprepared. But many profess to be incredulous as, to the information which percussion and auscultation can impart. Of such we may justly say, that they have no right—because they are not qualified—to judge, until they have made themselves *familiar* with the application of the hand and the stethoscope, both to the sound and the unsound thorax. If they *then* reject these measures of diagnosis, their testimony may be allowed its due weight. We are persuaded, however, that they will be too well satisfied with the results of their experience, to appear in opposition. It cannot be long, before they must meet with cases, that will afford them a gratification not unlike that which we have lately experienced from the following. A medical gentleman, of Tennesee, requested us to investigate his situation. He laboured under fever, strongly inclining to a hectic character, with cough, expectoration, and considerable emaciation. In short, he exhibited strong, *prima facie*, evidence, of a serious pulmonary læsion. On examining his chest, however, we were a good deal staggered. He not only bore thoracic pressure well, but it actually afforded him agreeable sensations; under percussion every spot emitted a hollow sound; the respiratory murmur could be heard in all parts; there was no rattle, no metallic tinkling, no ægophony, no pectoriloquism, and only that degree of broncophony, in the posterior and upper parts of the chest, which can be heard in most emaciated persons. From these observations, according to the new system of diagnosis, I was required to believe his lungs sound, although the signs of some kind of læsion were apparently unequivocal. At my next visit every difficulty was vanquished, by his informing me, that from the beginning, his larynx and trachea had been affected with tenderness, and a discharge of sputa. In fact, he laboured under a real trachitis, with cough and fever; and hence his lungs, when subjected to percussion and auscultation, gave no evidence of disordered structure. On the whole we believe that an experienced auscultator, may detect most of the organic changes to which the lungs are liable.

On this point there can be no difference of opinion among those who practice auscultation. There is however, some ground, on which to raise a doubt concerning the utility of this kind of diagnostic knowledge when acquired.

1. It relates to derangements of structure consequent upon disorders of function, which are not, in general, cognizable by the cylinder, and in most cases the organic læsions are irremediable.

2. Inflammation, either acute or chronic, is the morbid action which generates these structural derangements; and inflammation, in whatever tissue it may be located, generally requires the same *methodus medendi*; it would not, therefore, seem to be important, to know whether a pulmonary inflammation was seated in the mucous membrane, the serous membrane, or the intervening cellular tissue, or whether that tissue was becoming hepatized or tubercular.

Allowing to the whole of these arguments, what, in terms not very definite, we shall call a reasonable importance, we find nothing in them to justify a neglect of the measures which may lead to a correct diagnosis. Every kind of pathological knowledge must sooner or later, in some way or other, be useful in practice; but it is not true, that the stethoscope gives no *early* indication of organic changes. In the first stages of bronchitis and pleurisy, and while hæmoptysis or pulmonary apoplexy, is yet in its forming stage, the stethoscope *may* clear up many of our doubts; and impart a character of accuracy and science to our opinions, equally calculated to win the confidence of the sick, and advance the dignity of the profession. Is it a fact, however, that the result can in no degree be influenced by a knowledge of the tissue affected? We do not think it is. Every tissue has its proper functions, its appropriate sensibilities, and peculiar sympathies; and when inflamed, while certain things, required by all the phlegmasiæ, must be made the basis of our treatment, others, especially adapted to each variety, are not to be neglected. Thus in chronic pleurisy, cupping is a valuable remedy, while colchicum and balsam copaiva are useful medicines in bronchitis, and these methods are not convertible; in chronic pneumony the antiphlogistic treatment may arrest the progress of hepatization, but would be fruitless or prejudicial, if the lungs were tuberculated; in empyema, if the purulent collection should remain undiscovered, it might at length break into the bronchial ramifications and prove fatal, while, if the pus had been evacuated by paracentesis at an early period, the patient would have recovered. Other examples might be cited; but we shall submit the subject to the good sense of our readers, whose experiments and observations, we shall take great pleasure in presenting to the public.

## Figures and Tables

**Fig. 1. f1:**
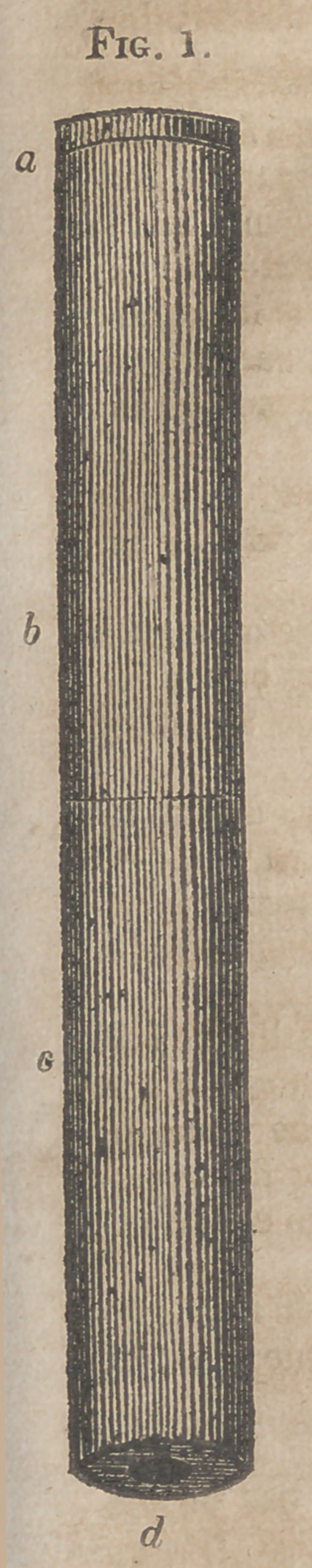


**Fig. 2. f2:**
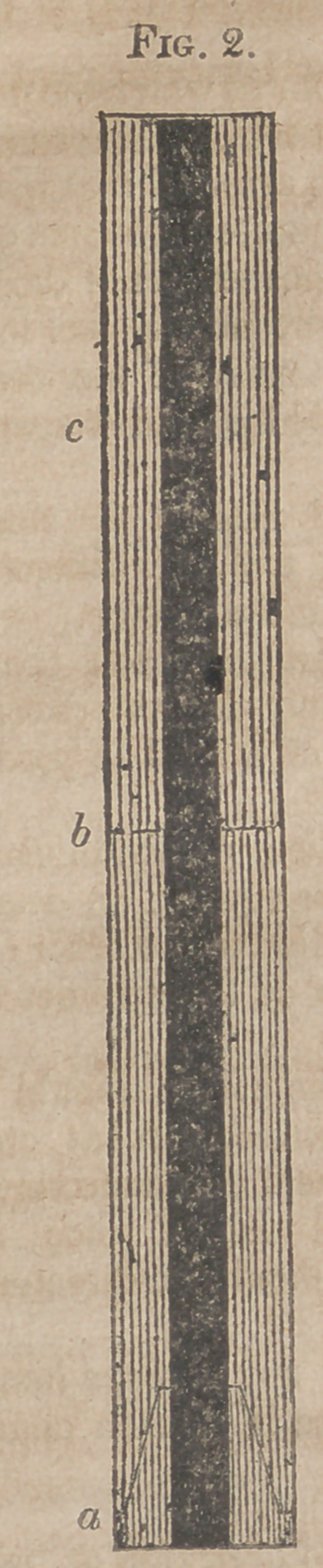


**Fig. 3. f3:**
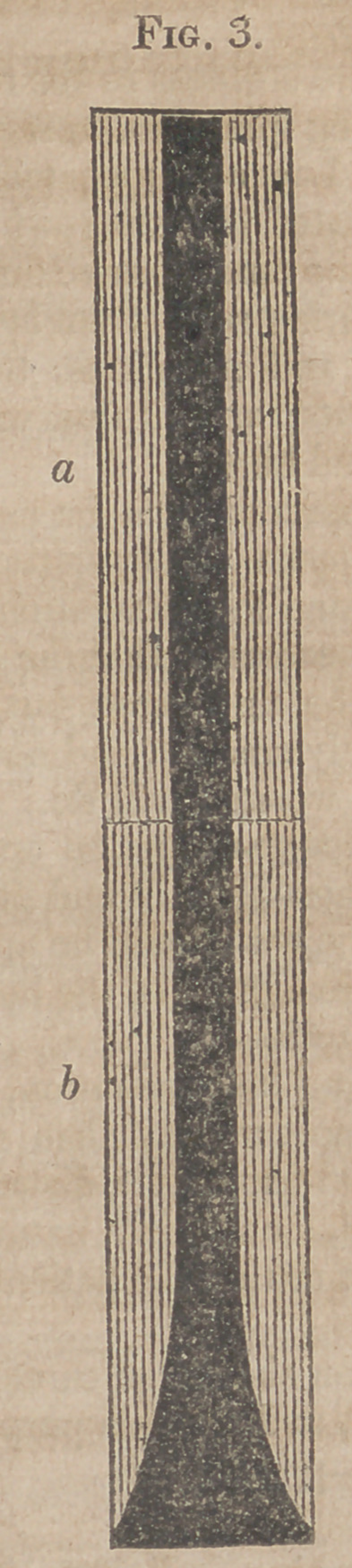


**Fig. 4. f4:**
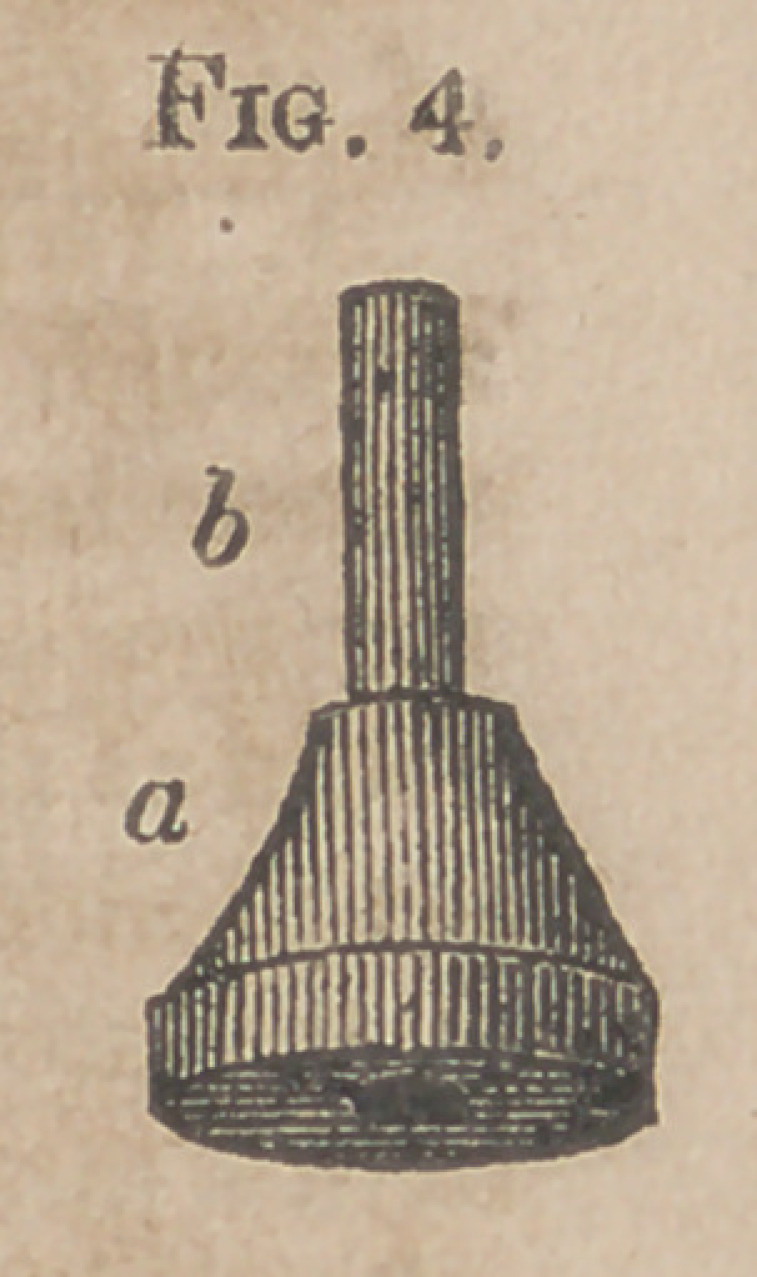


**Fig. 5. f5:**
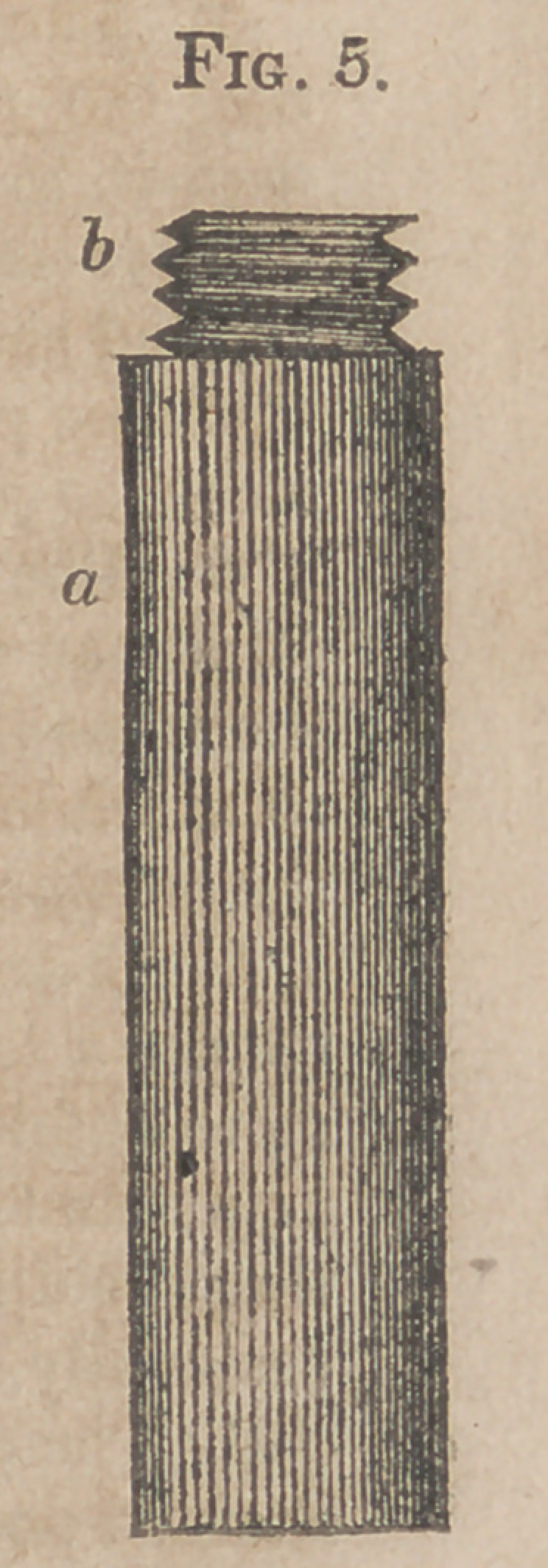


**Fig. 6. f6:**